# Development and validation of a model for stratifying haematological malignancy risk in primary care using basic blood tests

**DOI:** 10.1111/bjh.70798

**Published:** 2026-09-24

**Authors:** Mathilde Egelund Christensen, Michael Charles Sachs, William Grant Dunn, Gustav Jonzon, Margit Kriegbaum, Bent Struer Lind, Jan Samuelsson, Kirsten Groenbaek, George Vassiliou, Christen Lykkegaard Andersen

**Affiliations:** ^1^ Research Unit for General Practice, Department of Public Health University of Copenhagen Copenhagen Denmark; ^2^ Section of Biostatistics, Department of Public Health University of Copenhagen Copenhagen Denmark; ^3^ Wellcome‐MRC Cambridge Stem Cell Institute University of Cambridge Cambridge UK; ^4^ Department of Medical Epidemiology and Biostatistics Karolinska Institute Stockholm Sweden; ^5^ Department of Clinical Biochemistry Copenhagen University Hospital Hvidovre Denmark; ^6^ Department of Hematology Linköping University Hospital Linköping Sweden; ^7^ Department of Clinical Medicine, Faculty of Health and Medical Sciences University of Copenhagen Copenhagen Denmark

**Keywords:** biomarkers, early detection of cancer, haematology, machine learning, primary care

## Abstract

Haematological malignancies are rare in primary care and are hard to predict. Existing prediction models often require a specific working diagnosis and are based on extensive clinical information, access to which may be limited at initial work‐up in primary care. We aimed to develop a tool for estimating overall risk of haematological malignancy in primary care patients using simple data inputs. Including all full blood cell counts (FBCs) measured from 2000 to 2016 in 856 403 adult primary care patients in Eastern Denmark, we used machine learning to develop CBC‐HEMA, a risk assessment tool using FBCs, age, sex and C‐reactive protein measures to stratify ‘any haematological malignancy’ risk within 6 months, 1 year and 3 years with area under the curve (AUC) of 0.81, 0.78 and 0.71 respectively. Predictions of chronic lymphoid leukaemia and myeloproliferative disease were excellent, while CBC‐HEMA was unable to accurately predict non‐Hodgkin lymphoma and plasma cell dyscrasia. External validation was conducted in 123 015 primary care patients from Western Denmark with similar results and in 418 410 individuals from the background population cohort UK Biobank with inferior performance showing unfitness in screening healthy populations. The CBC‐HEMA https://shiny.sund.ku.dk/CopLab/blood‐cancer‐risk‐prediction/ may support risk assessment in primary care but cannot replace a clinical evaluation.

## INTRODUCTION

Haematological malignancies are rare in primary health care and most general practitioners (GPs) only encounter a few cases during their professional careers.^1^ Guidelines for referral from primary to specialist care differ among healthcare systems, but most recommendations are based on updated versions of the World Health Organization (WHO) diagnostic guidelines.^2^, ^3^, ^4^, ^5^ Applying these diagnostic guidelines as referral criteria in a primary care population is challenging, however, as several differential diagnoses are more prevalent in this context.^6^, ^7^ Relative risks of chronic leukaemia may be high with, for instance, eosinophilia or monocytosis, but absolute risks are still very low.^8^, ^9^

Risk prediction models for haematological malignancy are increasingly available, but, while several models appear quite accurate in their predictions, most lack external validation.^10^, ^11^, ^12^, ^13^, ^14^, ^15^, ^16^, ^17^ Their clinical implementation in primary care is limited, which could be attributed to two key factors: (1) most predict specific haematological malignancies and (2) most require numerous predictors including multiple biochemical analyses and molecular data.^10^, ^11^, ^12^, ^13^, ^14^, ^15^, ^16^, ^17^, ^18^ In primary care, a specific haematological diagnosis is not always suspected. Rather, the GP may need to opt for additional testing or specialist referral following only abnormal results from initial work‐up. Previous studies indicate the full blood cell count (FBC) is strongly associated with several haematological malignancies in the pre‐diagnostic phase, and the FBC is often available early in the diagnostic process.^8^, ^9^, ^15^, ^19^, ^20^, ^21^ Through advanced modelling, the FBC might predict haematological malignancy given a sufficiently large and unselected training dataset.

We aimed to develop and thoroughly validate a clinical triage tool based on machine learning, quantifying the risk of any haematological malignancy in primary care patients based on FBC counts, C‐reactive protein and demographics alone. The model should support the GPs' decision to refer the patient to specialist care without additional laboratory testing or a tentative haematological diagnosis.

## METHODS

### Methods in short

We split a large dataset of real‐world laboratory results from primary care in the general Copenhagen area into separate training and internal validation subsets, training a supervised stacked machine learning model to predict haematological malignancy within 6 months, 1 year and 3 years based on age, sex, FBC and C‐reactive protein (CRP). We then transformed it to an exportable tree‐based model, CBC‐HEMA. Model positive predictive value (PPV) was compared to simple laboratory cut‐offs as well as historic referrals for haematology. CBC‐HEMA was externally validated in a different primary care cohort from Western Denmark as well as in a cohort of healthy volunteers, UK Biobank, testing both reproducibility and transportability to a population screening scenario.

### Population

The Copenhagen Primary Care Laboratory Database (CopLab) contains all laboratory tests ordered by primary care physicians in the greater Copenhagen area from 2000 to 2015.^22^ We included adults (≥18 years) with at least one FBC recorded between 2006 and 2015 and Danish residence at inclusion. Individuals with prior haematological malignancy were excluded based on the Danish Cancer Register.^23^ To allow assessment of prior blood counts, FBCs from 2001 to 2005 were used only as historical data.

### Complete blood cell counts and C‐reactive protein measures

FBCs were analysed using Siemens (Bayer/Technicon, Munich, Germany) haematology systems. FBCs consisted of haemoglobin, platelets and leucocyte counts (no missing values) as well as up to 10 differential cell counts most often included on clinicians' request (see Supplementary Material for details).^22^, ^24^ CRP was measured in serum using the commercially available assays from Olympus A600 (Olympus A/S, Denmark) and ADVIA Chemistry System 1650/2400 (Bayer®/Siemens®, Denmark).^25^

### Outcomes—Danish nationwide registries

Haematological diagnoses were extracted from the Danish Cancer Register containing information on every Danish resident diagnosed with a malignant disease since 1943, with data currently available until 2021.^23^ Diagnoses were made by specialists in haematology, oncology and haematopathology following continuously updated international clinical guidelines^2^, ^3^, ^4^, ^5^ and then transferred directly from patient records to the register. We included diagnoses from 2006 to 2018 and grouped them into the major diagnostic subgroups of haematology (Table S2). These data were linked to the CopLab database via the Civil Registration Service, using the unique identification code, CPR (central persons register) issued for every Danish resident at birth or immigration, tracking information on date of death or emigration.^26^

### Building the model

The CopLab cohort was randomly split (60:40) into training and internal validation sets with complete separation of individuals. In the training set, FBCs obtained within 6 months, 1 year or 3 years before a diagnosis of haematological malignancy were identified, with death and emigration treated as competing events. For each case, five matched control events were sampled by age and sex. A stacked supervised machine learning ensemble was trained using demographic variables, FBC parameters (Table S1), CRP and calendar month. Because the case–control design inflated disease prevalence, the ensemble predictions were recalibrated in a subset of the internal validation cohort to reflect the true population incidence.

Due to data governance restrictions on exporting the trained ensemble model, we developed a tree‐based surrogate model using the same predictors and calibrated it to reproduce ensemble predictions enabling full external validation. This surrogate model, CBC‐HEMA, was used for all internal and external validations. Only results from the validation were included below, while no statistics were reported on the training data. Full details of model training and recalibration are provided in the Supplementary Material.

### External validation

External validation was done in two separate cohorts: (1) in real‐world data from Western Danish primary care, WDK and (2) in the UK Biobank (UKB).

WDK was formed using The Danish Clinical Laboratory Information Register^27^ containing results from all primary care blood tests from Jutland and Funen since 2015. We included individuals >18 years old at blood sampling, living in Jutland or Funen from 1 January 2016 to 31 December 2016. Laboratory data were merged with the Danish Cancer Register and Civil Registration Service for outcome assessment, and individuals with prior haematological disease were excluded forming the final WDK validation cohort. To estimate model transportability as a general screening tool, the UKB population was used for a second external validation. The UKB is a prospective volunteer cohort of ~500 000 healthy background population individuals aged 40–69 at inclusion and recruited from 2006 to 2010. Participants provided health data including biochemical measurements at baseline and were subsequently followed in the British health registers with data currently available until 2021.^28^ We included all UKB individuals, excluding participants due to prior haematological malignancy and missing outcome data.

### Statistical methods

The receiver operating characteristics (ROC) curves and areas under curve (AUC) for model predictions were made using the plotROC R statistical package^29^ assessing the ability to predict haematological malignancy within 6 months, 1 year and 3 years. As the 6‐month model had the best predictive performance, it was used for developing the surrogate model, CBC‐HEMA. The 6‐month ensemble model and the surrogate model, CBC‐HEMA were validated on the WDK data, while the surrogate model alone, CBC‐HEMA was validated on the UKB data. We present the results for the CBC‐HEMA model performance below, while AUCs for the full underlying ensemble model with and without FBC history may be found in the Supplementary Material. Calibration curves presented in Supplementary Material were used to compare CBC‐HEMA and the ensemble model calibration in CopLab and WDK.

We calculated PPVs for the CBC‐HEMA across all probability thresholds, presenting a PPV‐curve for the 6‐month predictions in CopLab, WDK and UKB.

We tested the model performance in predicting specific haematological disease groups, comparing ROC curves and AUCs for the 6‐month, 1‐year and 3‐year model predictions within each diagnostic subgroup (Table S2). We evaluated and ranked the features of the model most important in predicting each diagnostic subgroup, presenting the five most important features for each diagnosis. We plotted the distribution of the model's predicted risks according to the observed diagnoses.

We compared model predictions with historic referrals for haematology by including data from the National Patient Register^30^ on outpatient clinic visits in haematology and clinical quality assurance data on rejected referrals; national referral guidelines continuously mirrored the updated WHO recommendations.^2^, ^3^, ^31^, ^32^ These data were coupled with cancer outcome data to estimate sensitivity, specificity and PPV (Supplementary Material) of current standard of care for comparison with model predictions. To compare the model predictions with the performance of simple laboratory cut‐offs, we used laboratory values referenced in the WHO 2022 and 2016 guidelines (Table S3).^2^, ^3^, ^33^, ^34^ To further illustrate clinical utility, we used decision curves comparing the net benefit of CBC‐HEMA versus historic referrals and the WHO 2022 and 2016 laboratory cut‐offs.^35^

## RESULTS

We included 879 110 adults (*N* = 3 569 268 CBCs) with at least one FBC registered in the CopLab database between 1 January 2006 and 31 December 2015. We excluded individuals with prior haematological malignancy and with missing values of haemoglobin, platelets and leucocytes, leaving 856 403 individuals (*N* = 3 409 381 FBCs) for our final study population. Participants had a median age of 47 years, and the incidence of haematological malignancy was 105 per 100.000 person years at risk (Table 1, Figure 1).

**TABLE 1:** Basic characteristics.

	CopLab		WDK		UKB	
	*N* = 855 529		*N* = 123 015		*N* = 418 410	
		% missing		% missing		% missing
Sex						
Male	42.4%	0%	43.6%	0%	45.3%	0%
Female	57.6%	0%	56.4%	0%	54.7%	0%
Age, years	47.0 [31.0–61.0]	0%	63.2 [49.1–74.1]	0%	58.0 [51.0–64.0]	0%
Haemoglobin, mmol/L	8.6 [8.0–9.2]	0%	8.6 [8.0–9.2]	0%	8.5 [8.0–9.0]	0%
Platelets, *n* × 10^9^	271 [230–319]	0%	255 [214–303]	0%	248 [213–288]	0%
Leucocytes, *n* × 10^9^	7.00 [5.80–8.60]	0%	6.80 [7.70–8.30]	0%	6.69 [5.67–7.90]	0%
Lymphocytes	1.99 [1.60–2.46]	46.8%	1.91 [1.51–2.38]	65.3%	1.89 [1.51–2.30]	0%
Neutrophils	3.99 [3.07–5.26]	46.8%	4.02 [3.09–5.29]	64.4%	4.05 [3.30–5.00]	0%
Monocytes	0.41 [0.33–0.52]	46.8%	0.50 [0.39–0.64]	65.3%	0.45 [0.37–0.57]	0%
Eosinophils	0.17 [0.11–0.27]	46.8%	0.16 [0.10–0.25]	62.1%	0.14 [0.10–0.21]	0%
Basophils	0.04 [0.02–0.05]	46.8%	0.04 [0.03–0.06]	65.3%	0.012 [0.00–0.04]	0%
Myelocytes	0.00 [0.00–0.00]	46.8%	0.00 [0.00–0.00]	64.4%	NA	100%
Metamyelocytes	0.00 [0.00–0.00]	46.8%	0.00 [0.00–0.00]	64.4%	NA	100%
Promyelocytes	0.00 [0.00–0.00]	46.8%	0.00 [0.00–0.00]	64.4%	NA	100%
Blasts	0.00 [0.00–0.00]	46.8%	0.00 [0.00–0.00]	64.4%	NA	100%
Unclassifiable	0.14 [0.11–0.19]	46.8%	NA	100%	NA	100%
C‐reactive protein, mg/L	5.0 [4.0–5.0]	37.5%	2.90 [49.2%]	49.2%	1.37 [0.67–2.85]	0%
Cancer at 6 months	0.14%	0%	0.3%	0%	0.0%	13.5%
Cancer at 1 year	0.19%	0%	0.4%	0%	0.1%	13.5%
Cancer at 3 years	0.34%	0%	0.8%	0%	0.3%	13.5%
Mortality, 6 months	1.01%	0%	0.5%	0%	0.1%	13.5%
Mortality, 1 year	1.78%	0%	1.7%	0%	0.2%	13.5%
Mortality, 3 years	4.48%	0%	7.6%	0%	0.9%	13.5%

*Note*: Unclassifiable = unclassifiable cells detected in the automated full blood cell count; manual count performed. Median and [Q1–Q3] for continuous variables and percent for categorical. Predictor variables consisting as well as outcome measures including incident haematological malignancy and mortality within 6 months, 1 year and 3 years for the CopLab cohort as well as two external validation cohorts; CopLab, consisting of primary care patients from Eastern Denmark registered with a FBC from 2006 to 2015 showing characteristics for internal validation subset. WDK, an external validation cohort consisting of primary care patients from Western Denmark through 2016. UKB, an external validation cohort consisting of volunteers from the British background population, 2006–2010.

Abbreviations: CopLab, Copenhagen Primary Care Laboratory Database; UKB, UK Biobank; WDK, Western Denmark, primary care population.

**FIGURE 1:**
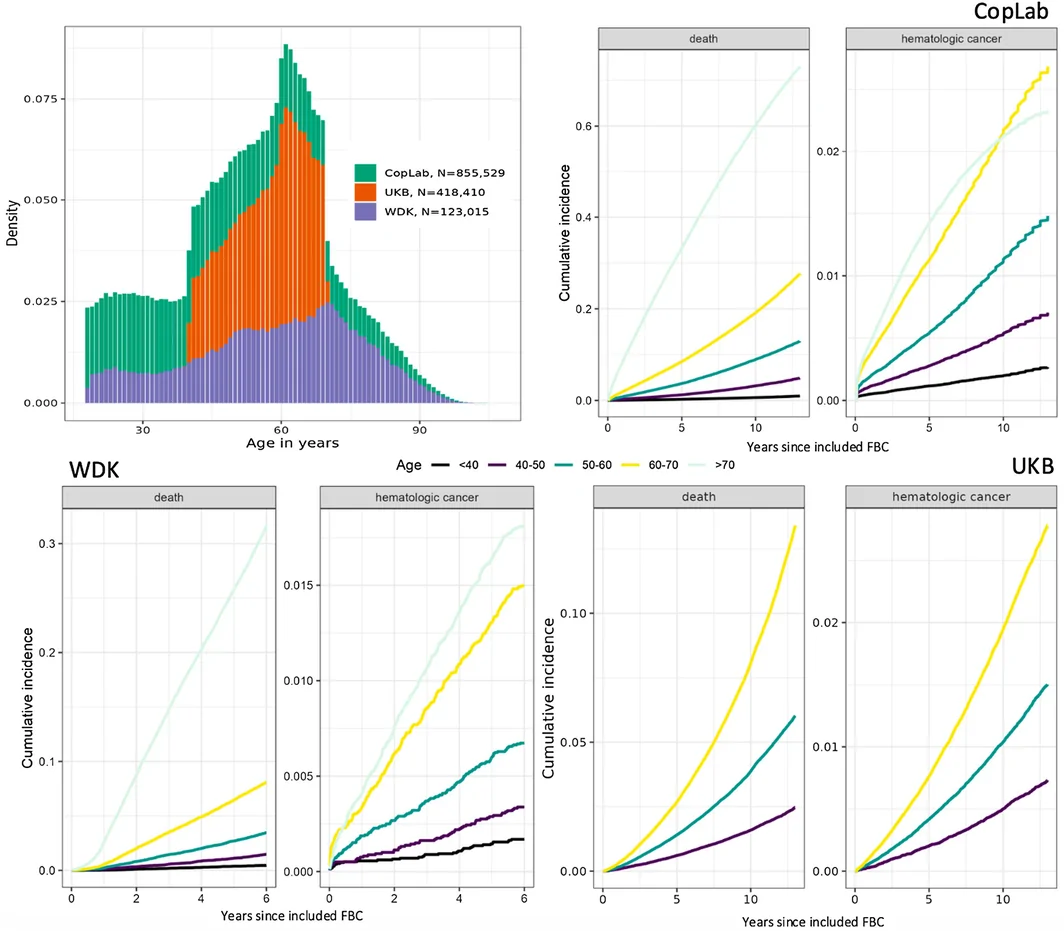
Age distribution and cumulated incidence of mortality and haematological malignancy in CopLab, WDK and UKB. CopLab = Copenhagen Primary Care Laboratory Database. WDK = Western Denmark. UKB = UK Biobank. FBC = full blood cell count. A: Histogram of age distribution in the combined CopLab cohort separate subsets of which were used to train and internally validate the model and in external validation cohorts: WDK consisting of primary care patients from Western Denmark and UKB consisting of volunteers from the British background population. B: Cumulated incidence of haematological malignancy and death in CopLab, WDK and UKB. NB: Axes vary due to very small numbers in UKB. For a version of the figure with fixed axes, see Supplementary Material.

### Predictive performance

CBC‐HEMA predicting overall haematological malignancy in CopLab within 6 months, 1 year and 3 years yielded AUCs of 0.81 (95% confidence interval [CI] 0.80–0.83), 0.78 (95% CI 0.77–0.79) and 0.71 (95% CI 0.70–0.72) respectively (Figure 2). Calibration curves (Figure S1) indicated a mild underestimation of risk, although calibration was generally acceptable.

**FIGURE 2:**
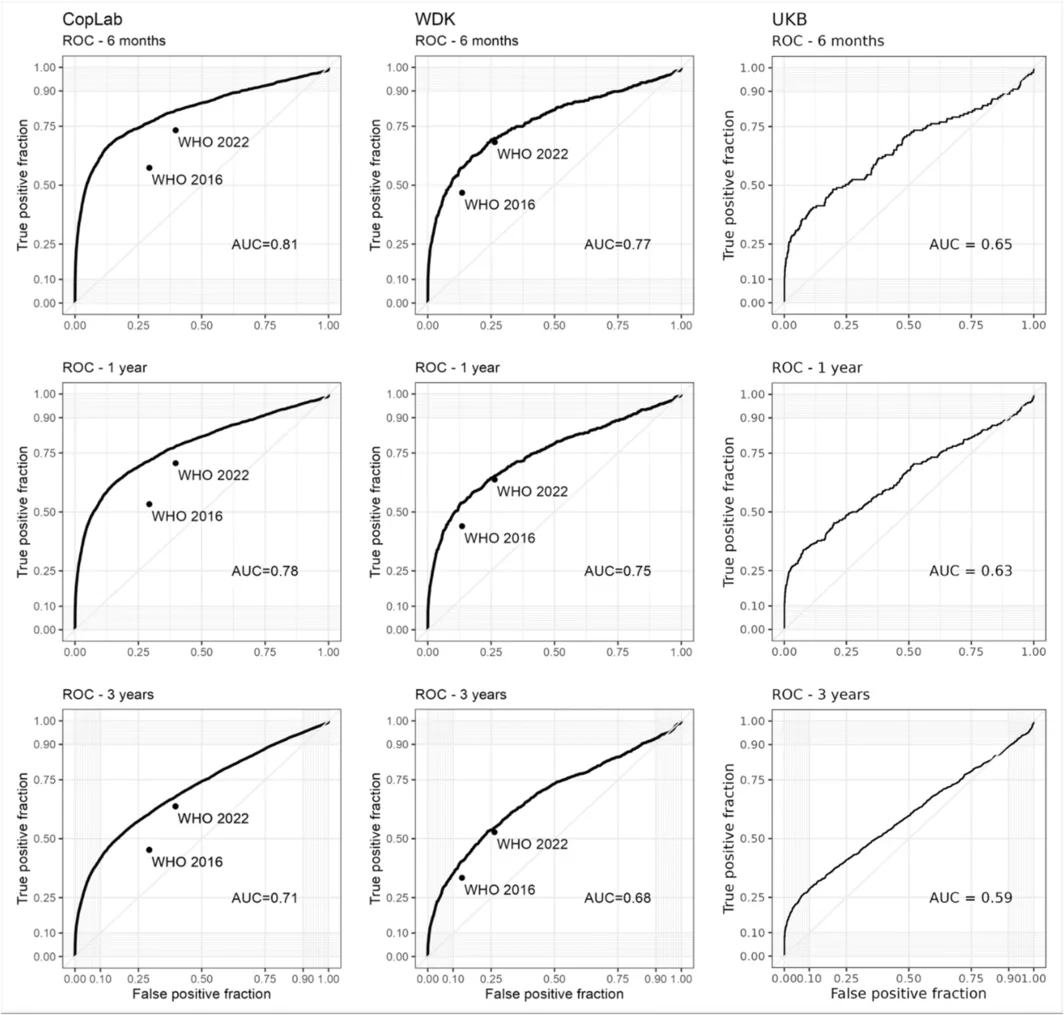
Receiver Operating Characteristic Curves for predicting risk of any haematological malignancy in CopLab, WDK and UKB; CopLab = Copenhagen Primary Care Laboratory Database. WDK = Western Denmark. UKB = UK Biobank. ROC = Receiver Operating Characteristic. WHO 2016 and 2022 = performance of simple laboratory cut‐offs based on the WHO diagnostic guidelines from 2016 and 2022 (Table S3). Receiver operating characteristic (ROC) curves for predicting any haematological malignancy within 6 months, 1 year and 3 years following a FBC in the internal validation cohort from CopLab and in external validation cohorts: WDK consisting of primary care patients from Western Denmark and UKB consisting of volunteers from the British background population. The CopLab cohort used to train the model was kept separate from the internal validation cohort; model performance in the training subset is not reported.

The most precise predictions were accomplished in the 6‐month risk of chronic lymphocytic leukaemia (CLL) and myeloproliferative neoplasms (MPN), whereas the model was less capable of predicting non‐Hodgkin lymphoma (NHL) and plasma cell neoplasms (Table 2, Figure S2). The models' predictive performance attenuated over time for all diagnostic subgroups, but only minimally for CLL, MDS and MPN, where 3‐year predictions almost matched the 6‐month results.

**TABLE 2:** Area under the receiver operating characteristics curve for predicting specific haematological malignancies in CopLab, WDK and UKB.

Cohort	Time horizon	MDS	MPN	AML	ALL	CLL	Hodgkin	NHL	PCD
CopLab	6 months	0.83	0.91	0.82	0.78	0.93	0.80	0.70	0.73
*N* = 855 529	1 year	0.82	0.90	0.73	0.69	0.91	0.79	0.66	0.70
	3 years	0.77	0.85	0.63	0.60	0.85	0.69	0.60	0.63
WDK	6 months	0.84	0.80	0.81	0.48	0.92	0.76	0.67	0.74
*N* = 123 015	1 year	0.83	0.80	0.78	0.52	0.90	0.75	0.64	0.70
	3 years	0.76	0.74	0.62	0.60	0.79	0.68	0.57	0.66
UK Biobank	6 months	0.69	0.61	0.49	0.41	0.95	0.87	0.61	0.65
*N* = 418 410	1 year	0.65	0.67	0.54	0.41	0.91	0.74	0.58	0.57
	3 years	0.58	0.57	0.52	0.62	0.85	0.57	0.55	0.58

*Note*: Showing the area under curve for receiver operating characteristics curves for predicting specific haematological malignancy within 6 months, 1 year and 3 years for the CopLab cohort as well as two external validation cohorts; CopLab, consisting of primary care patients from Eastern Denmark registered with a full blood cell count from 2006 to 2015. WDK, an external validation cohort consisting of primary care patients from Western Denmark through 2016. UKB, an external validation cohort consisting of volunteers from the British background population, 2006–2010. Specific diagnoses included in each disease group are specified in Table S2.

Abbreviations: CLL, chronic lymphoid leukaemia; CopLab, Copenhagen Primary Care Laboratory Database; HM, haematological malignancy; Hodgkin, Hodgkin lymphoma; MDS, myelodysplastic neoplasms; MPN, myeloproliferative neoplasms; NHL, non‐Hodgkin lymphoma; PCD, plasma cell disease; UKB, UK Biobank; WDK, Western Denmark, primary care population.

Patients later diagnosed with CLL, MDS and MPN had the highest predicted risks, while predicted risks were generally lower for patients who developed lymphoma or acute leukaemia (Figure 3A). Lymphocyte count, total leucocyte count and monocyte count were the most important variables in predicting CLL, while platelet count, neutrophil count and haemoglobin were most predictive in MPN. Age and neutrophil count were predictive of Hodgkin lymphoma, and the presence of blasts was predictive only of AML (Figure 3B).

**FIGURE 3:**
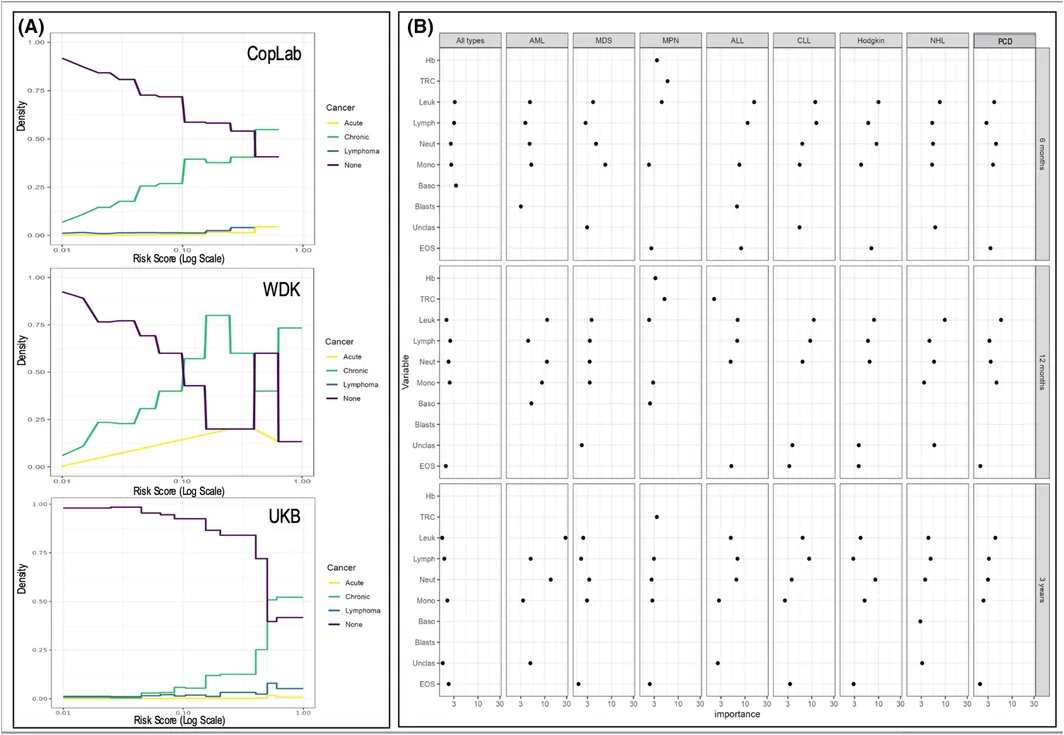
Distribution of risk scores in the CopLab, WDK and UKB populations and variables most important for predicting each diagnosis (A) CopLab = Copenhagen Primary Care Laboratory Database. WDK = Western Denmark. UKB = UK Biobank. Acute = acute myeloid leukaemia and acute lymphoblastic leukaemia. Chronic = myeloproliferative neoplasms, myelodysplastic syndromes and chronic lymphocytic leukaemia. Lymphoma = Hodgkin lymphoma, non‐Hodgkin lymphoma and plasma cell disorders. None = no malignant haematological diagnosis. Distribution of diagnoses for each risk score for predicting any haematological malignancy within 6 months in the internal validation cohort from CopLab and in external validation cohorts: WDK consisting of primary care patients from Western Denmark and UKB consisting of volunteers from the British background population. (B) CLL = chronic lymphoid leukaemia. PCD = plasma cell disease. Hodgkin = Hodgkin lymphoma. NHL = non‐Hodgkin lymphoma. Hb = Haemoglobin, TRC = platelets, Leuk = total leucocytes, Lymph = lymphocytes, Neut = neutrophils, Mono = monocytes, Baso = basophils, UNCLAS = unclassifiable cells detected in the automated complete blood cell count, manual count performed. The plot shows the five most important variables for predicting each subgroup of haematological malignancy within 6 months, 12 months and 3 years of the full blood cell count in the internal validation cohort from CopLab.

### Comparison with standard of care

Every 3 months, 0.12% of CopLab participants were referred to a haematologist. Of these referrals, 61% led to actual visits and 39% were rejected by a haematologist before a visit. At the haematological clinics, 26% of the patients investigated received a diagnosis, resulting in a PPV of 21% for being diagnosed with a haematological malignancy after being referred from primary care (detailed calculations in Supplementary Material). CBC‐HEMA PPVs were inferior to historic referrals for threshold probabilities below 1.5% and superior for the range of thresholds above this level (Figure 4). Simple laboratory cut‐offs represented by criteria from the WHO 2022 and 2016 guidelines (Table S3) were on average violated in 38% and 29% of CopLab participants, respectively, every 3 months resulting in PPVs of under 0.3% for predicting haematological malignancy (Table S3, Figure 4).^2^, ^3^, ^33^, ^34^ Decision curve analysis suggested superiority for CBC‐HEMA predictions compared with the laboratory cut‐offs. For threshold probabilities below 10%, CBC‐HEMA appeared to represent an increased net benefit compared with historic referrals, while the threshold probability had to exceed 25% to match the net benefit for referring no patients (Figure 5).

**FIGURE 4:**
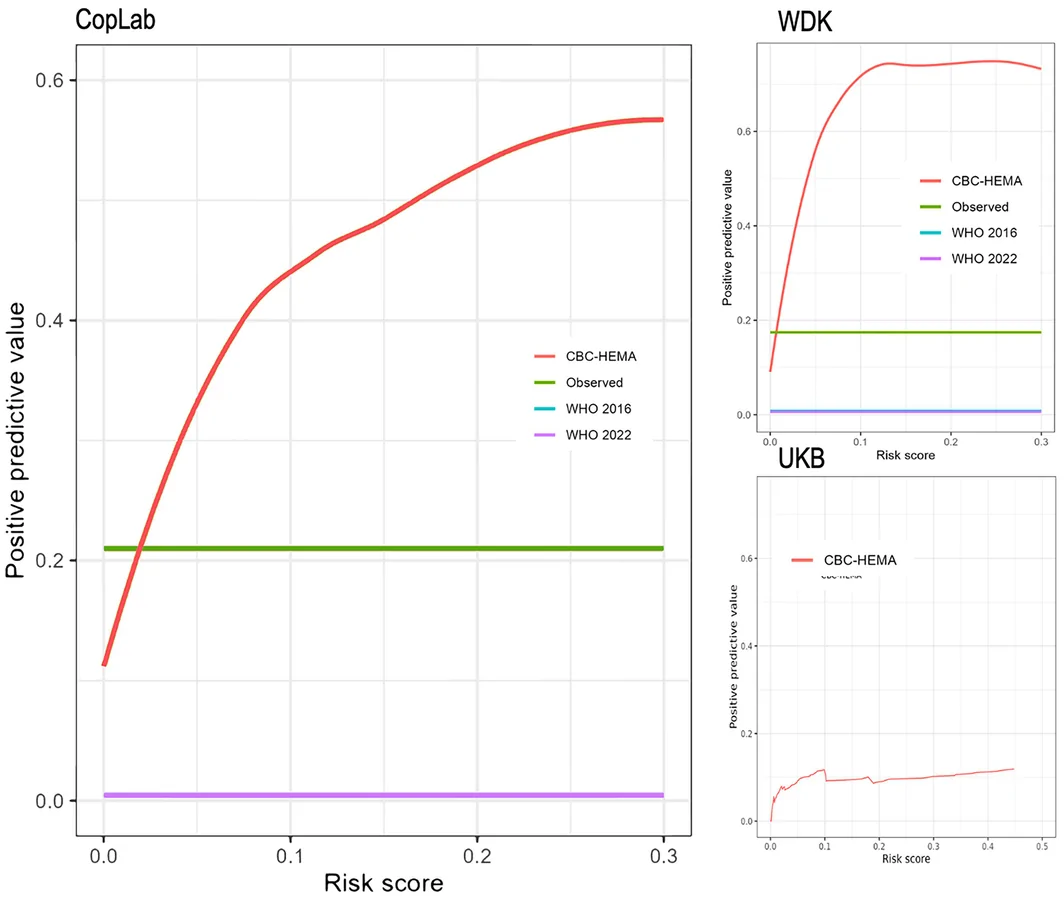
Prediction curves for CopLab, WDK and UKB. CopLab = Copenhagen Primary Care Laboratory Database. WDK = Western Denmark. UKB = UK Biobank. PPV = positive predictive value. Observed = historic data on referrals from primary to secondary care (department of haematology). WHO 2016 and 2022 = performance of simple laboratory cut‐offs based on the WHO diagnostic guidelines from 2016 and 2022. Prediction curves for predicting any haematological malignancy within 6 months following a FBC showing the positive predictive value for each risk score between 0 and 1 predicted by the model in the internal validation cohort from CopLab and in external validation cohorts: WDK consisting of primary care patients from Western Denmark and UKB consisting of volunteers from the British background population. The CopLab cohort used to train the model was kept separate from the internal validation cohort; model performance in the training subset is not reported.

**FIGURE 5:**
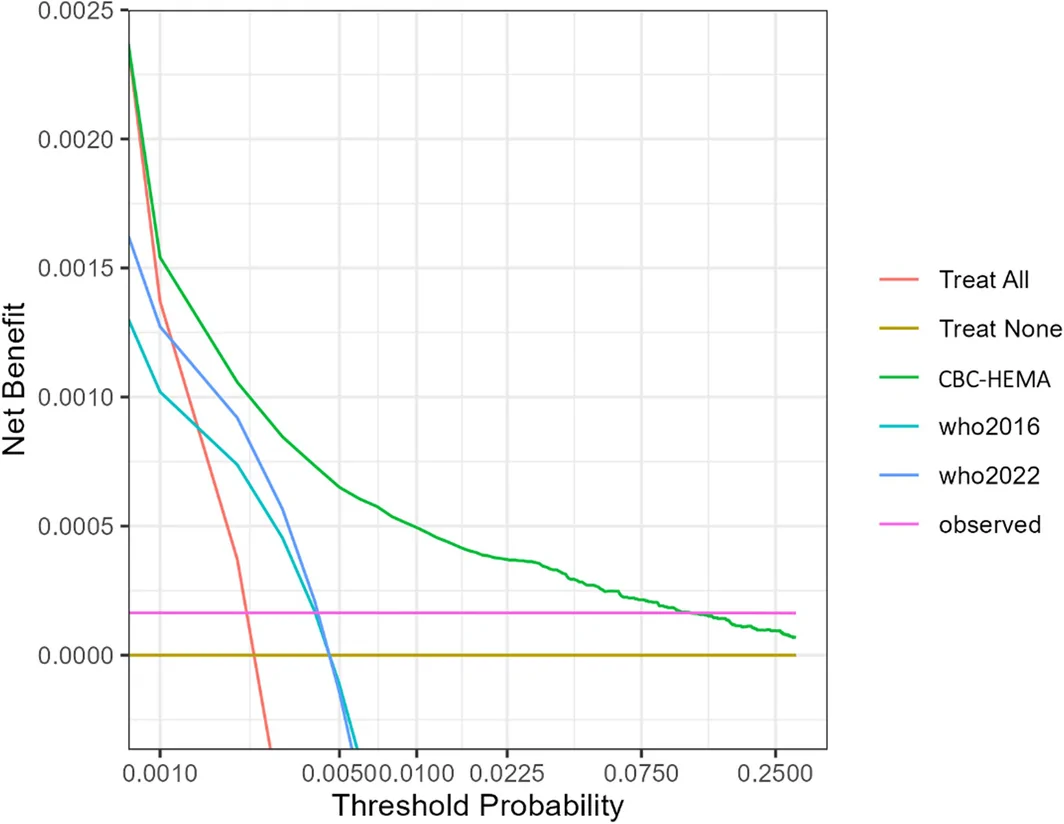
Decision curve analysis for CopLab and WDK Observed = historic data on referrals from primary to secondary care (department of haematology). WHO 2016 and 2022 = performance of simple laboratory cut‐offs based on the WHO diagnostic guidelines from 2016 and 2022. Decision curves illustrating the net benefit of the CBC‐HEMA prediction model compared with simple laboratory cut‐offs and historic referral data. Net benefit = TPF ‐ FPF * (p/(1 − p)), where TPF = true‐positive fraction, FPF = false‐positive fraction, (p/(1 − p)) = relative harm of false positive to false negative.

### External validation

We included 123 015 adult individuals (*N* = 188 582 FBCs) with no prior haematological malignancy and no missing values of haemoglobin, platelets and leucocytes from the WDK cohort and 418 410 individuals (*N* = 418 410 CBCs) with a FBC in UKB after excluding 2204 individuals due to prior haematological malignancy and 56 693 due to missing outcome data (Table 1). The CopLab and WDK populations were characterized by wide age distributions including individuals above 80 years of age reflecting a primary care setting, contrasting with a narrower age interval in UKB reflecting UKB inclusion criteria (Table 1, Figure 1A). Cumulative incidences of haematological malignancy and mortality were unsurprisingly higher in CopLab and WDK than in UKB across all age groups (Figure 2B).

In WDK, CBC‐HEMA predicted overall haematological malignancy with AUCs comparable to CopLab results while inferior AUCs were observed in UKB (Table 2). As in CopLab, 6‐month risk of CLL and MPN showed the best performance and predictive power persisted over time (Figure S2). WDK PPVs were comparable to CopLab results, whereas UKB PPVs were poor (Figure 4).

## DISCUSSION

Using a population‐based cohort of 856 403 individuals from primary care, we developed and validated a model entitled CBC‐HEMA using FBC, CRP, age and sex for predicting any haematological malignancy within 6 months, 1 year and 3 years. The broad outcome definition and simple data input requirements were chosen to enable use at the initial clinical assessment, assisting the GP in identifying patients for referral to haematology without additional laboratory testing. The model was generally well calibrated but appeared to mildly underestimate risk. Comparison with historic referrals using PPV curves and the decision curve analysis indicated a range of threshold probabilities (1.5% to 10%), where CBC‐HEMA could benefit clinical decision‐making. All analyses suggested that the model was superior to simple laboratory cut‐offs across threshold probabilities.

In predicting ‘any haematological malignancy’, CBC‐HEMA approximates a previous regression model for predicting solid tumours in primary care by Hippisley‐Cox et al., who reported AUCs between 0.82 and 0.92 for individual cancer7 including medical and family history, smoking and alcohol consumption and clinical symptoms. CBC‐HEMA matches this performance with a much simpler predictor set, which is noticeable and most likely attributable to the stacking of models and machine learning methodology. However, CBC‐HEMA discriminatory power varied greatly across diagnoses: high performance was achieved for CLL, MDS and MPN, while CBC‐HEMA was effectively unable to discriminate paraprotein‐related diseases and NHL. Previous models by Koshiaris et al. for predicting myeloma in primary care had an AUC of 0.84/0.87 based on clinical symptoms, FBC measures and erythrocyte sedimentation rate, which was superior to CBC‐HEMA.^13^, ^14^ In NHL, PPVs of 13% were reported by Shepard et al. based on lymphadenopathy and age >60 years.^16^ These models were mainly separated from CBC‐HEMA by the inclusion of clinical symptoms, which are unsurprisingly highly predictive for myeloma and NHL. Lymphoma and paraprotein‐related diseases only occasionally influence FBC measures in the pre‐diagnostic stages, and this present study suggests that, even with advanced machine learning‐based methods, FBCs may be too unspecific for detecting these malignancies.^19^, ^36^, ^37^, ^38^ Conversely, CBC‐HEMA was on par with AML predictions achieved by Abelson et al. who trained several models based on age, sex, 50 laboratory measures and mutational profiling arriving at AUCs of 0.76–0.82.^15^

We externally validated CBC‐HEMA in 123 015 primary care individuals from Western Denmark where the predictive performance mirrored the internal validation, while external validation in 418 410 UKB individuals showed lower predictive performance overall, with particular deficit in AML predictions.^28^, ^39^ This may be partially explained by the healthy volunteer effect being stronger at the time of blood sampling as UKB FBCs were only measured at baseline.^39^ Screening for rare diseases in healthy individuals runs the risk of overdiagnosis and the inferior predictions in UKB emphasize that CBC‐HEMA is not transportable as a general screening tool. Should the model be used for population screening, it would require recalibration or retraining in a background population dataset.

CBC‐HEMA performance varied across diagnoses making it unsuitable for predicting any haematological malignancy, but its high discriminatory performance in MPN and CLL suggests that CBC‐HEMA in its current form could support GPs in stratifying patients with elevated complete blood cell counts for referral or additional testing. The calibration curve and decision curve analysis indicate that CBC‐HEMA predictions cannot replace a clinical evaluation. Comparison with previous regression models suggests that a machine learning approach can achieve high performance with a much simpler predictor set. The approach could be used in future studies, where models should include clinical symptoms registered at initial clinical assessment.

### Strengths and limitations

Strengths include the large sample size and population‐based design of the CopLab cohort, the linkage with exhaustive Danish registers including reliable outcome data on cancer events and death^23^, ^24^ and the homogenous assessment of predictor and outcome variables for all included patients. Strengths in the analyses include complete separation of training and internal validation subsets and multiple methods for dealing with missing data in the model training. External validation in two large cohorts shows generalizability to other primary care settings and comparison with standard of care PPV and decision curve analyses enable assessment of clinical utility. Limitations include the absence of primary care clinical observations. The inclusion of more laboratory measures with known predictive and/or diagnostic value such as red cell distribution width, erythrocyte sedimentation rate and plasma viscosity could potentially improve predictions,^13^, ^14^, ^15^, ^40^, ^41^ but these were omitted due to limited use in Danish primary care.

The inclusion of blood sampling season as a predictor necessitates a recalibration of the model before use in the southern hemisphere. The model was not validated for use in children or for predicting the risk of secondary haematological malignancy.

## CONCLUSION

We developed CBC‐HEMA, a model for stratifying primary care patients at risk of any haematological malignancy using full blood cell count (FBC) measures, CRP, age and sex. Trained and validated on data from over 800 000 individuals in Eastern Danish primary care and externally validated with >120 000 individuals from Western Danish primary care and >400 000 from the UK Biobank, the model showed high accuracy for CLL and MPN but failed to predict NHL and plasma cell dyscrasia. It outperformed simple laboratory cut‐offs and appeared to represent an increased clinical benefit in decision curve analysis particularly for low to moderate risk patients. CBC‐HEMA could aid clinical decision‐making in primary care patients with elevated blood cell counts but cannot guide referral independent of clinical assessment. Information on clinical symptoms could help improve predictions in machine learning models developed in the future. Access the calculator here: https://shiny.sund.ku.dk/CopLab/blood‐cancer‐risk‐prediction/.

## AUTHOR CONTRIBUTIONS

**Margit Kriegbaum:** Conceptualization; methodology; software; validation; investigation; data curation; resources; writing – review and editing. **Bent Struer Lind:** Conceptualization; methodology; investigation; resources; writing – review and editing. **Mathilde Egelund Christensen:** Conceptualization; methodology; validation; project administration; visualization; writing – original draft; writing – review and editing. **Michael Charles Sachs:** Conceptualization; methodology; software; validation; formal analysis; data curation; writing – original draft; writing – review and editing; visualization; project administration. **Jan Samuelsson:** Methodology; writing – review and editing. **Kirsten Groenbaek:** Conceptualization; writing – review and editing; funding acquisition; supervision. **Gustav Jonzon:** Formal analysis; data curation; writing – review and editing. **Christen Lykkegaard Andersen:** Conceptualization; methodology; validation; investigation; resources; project administration; supervision; funding acquisition; writing – review and editing. **George Vassiliou:** Investigation; supervision; visualization; resources; writing – review and editing. **William Grant Dunn:** Software; validation; data curation; formal analysis; visualization; writing – review and editing.

## FUNDING INFORMATION

W.G.D is funded by a Clinical Research Fellowship from the Cancer Research UK Cambridge Centre (CTRQQR‐2021\100012). G.S.V. was supported by a Cancer Research UK Senior Cancer Fellowship (C22324/A23015). The UKB‐related work was supported by an Early Detection Project Grant from Cancer Research UK (EDDCPJT\100010) and a joint grant from the Leukaemia and Lymphoma Society (RTF6006‐19). The Cambridge Stem Cell Institute is supported by the Wellcome Trust (203 151/Z/16/Z, 203151/A/16/Z) and the UKRI Medical Research Council (MC_PC_17230). C.L.A. obtained funding from the Danish Cancer Society (R327‐A18965). No funders were involved in the collection, analysis or interpretation of data nor did they have access to the data. No funders were involved with the writing of the manuscript or the decision to submit the article for publication.

## CONFLICT OF INTEREST STATEMENT

All authors have completed the ICMJE uniform disclosure form and declare: no support from any organization for the submitted work; no financial relationships with any organizations that might have an interest in the submitted work in the previous 3 years; no other relationships or activities that could appear to have influenced the submitted work.

## ETHICS STATEMENT

The CopLab database and its activities are approved by the Danish Data Protection Agency through the joint notification of The Faculty of Health and Medical Sciences at the University of Copenhagen (journal no. 2015‐57‐0121). According to Danish legislation, no ethical approval or patient consent was required because the patients were not approached at any time during the conduct of the study. No data were reproduced from previous work by the authors or others. UKB is a large‐scale biomedical database and research resource containing genetic, lifestyle and health information from half a million UK participants. UKB has approval from the North West Multicentre Research Ethics Committee (11/NW/0382) and all participants provided written informed consent. The present study has been conducted under approved UKB application number 56844. Electronic health records were downloaded from the UKB portal in April 2022.

## TRANSPARENCY

The lead author affirms that this manuscript is an honest, accurate and transparent account of the study being reported; that no important aspects of the study have been omitted; and that any discrepancies from the study as planned have been explained.

## Supporting information


**Table S1:** Catalogue of biochemical analyses included in the complete blood cell count in the CopLab database.
**Table S2:** ICD‐10 diagnoses included within each diagnostic subgroup.
**Table S3:** WHO 2022 and WHO 2016 laboratory criteria included for comparison.
**Figure S1:** Calibration curves for predicting any haematological malignancy.
**Figure S2:** Receiver operating characteristic curves for predicting risk of specific haematological diagnoses in CopLab, WDK and UKB within 6 months, 1 year and 3 years.

## Data Availability

The supplementary materials made available with publication of this study include the R‐script for the tree‐based surrogate model as well as a detailed methods section and Tables S1–S4. The Copenhagen Primary Care Laboratory Database (CopLab) is administered by the Department of Public Health, University of Copenhagen and access to CopLab data is governed by the CopLab steering group. Data cannot be made publicly available as it contains personal information. However, researchers irrespective of nationality and affiliation may submit applications which will then be assessed by the Steering Group of CopLab for data access (https://publichealth.ku.dk/research/databases‐for‐collaboration/coplab/).
