# Supplementary material for: Development and validation of a model for stratifying haematological malignancy risk in primary care using basic blood tests
Source: Br J Haematol. 2026 Sep 24;209(5):2044–55. doi: 10.1111/bjh.70798 (PMC13748387; doi:10.1111/bjh.70798)
Supplement: Supplementary file 1 [file BJH-209-2044-s001.docx]

[Supplementary methods	3](#_Toc234611999)

[Primary care population from Eastern Denmark (CopLab)	3](#_Toc234612000)

[Complete Blood Cell Counts	3](#_Toc234612001)

[Statistical methods: model building	3](#_Toc234612002)

[Sample splitting and exclusions	3](#_Toc234612003)

[Figure: Flow chart of sample splitting for model training and internal validation	6](#_Toc234612004)

[Model training	6](#_Toc234612005)

[Model evaluation	7](#_Toc234612006)

[Recalibration	7](#_Toc234612007)

[Surrogate model estimation	8](#_Toc234612008)

[Feature importance	8](#_Toc234612009)

[External validations	8](#_Toc234612010)

[Cumulative incidence according to predicted risk	8](#_Toc234612011)

[Statistical methods: comparison with standard of care	9](#_Toc234612012)

[Model – true positives, false positives and predictive values	9](#_Toc234612013)

[Standard of care – true positives, false positives and predictive values	9](#_Toc234612014)

[Results	11](#_Toc234612015)

[Performance of the full ensemble model	11](#_Toc234612016)

[Risk stratification using CBC-HEMA	12](#_Toc234612017)

[Figure: Cumulative incidence of any type of haematological malignancy by predicted risk score in the CopLab, WDK-and UKB-populations	13](#_Toc234612018)

[Table: ICD-10 codes for co-morbidity	14](#_Toc234612019)

[Sensitivity, Specificity, Positive and Negative Predictive Values	15](#_Toc234612020)

[Figure: Number of diagnoses and complete blood cell counts in CopLab by calendar year	16](#_Toc234612021)

[Figure: Cumulative incidence of haematological malignancy with fixed axes.	17](#_Toc234612022)

[References	18](#_Toc234612023)

[R code for the model	20](#_Toc234612024)

[Supplementary Table 1: Catalogue of biochemical analyses included in the complete blood cell count in the CopLab database	22](#_Toc234612025)

[Supplementary Table 2: ICD-10 diagnoses included within each diagnostic subgroup	23](#_Toc234612026)

[Supplementary Table 3: WHO 2022 and WHO 2016 laboratory criteria included for comparison	24](#_Toc234612027)

[Supplementary Figure 1: Calibration curves for predicting any haematological malignancy	25](#_Toc234612028)

[Supplementary Figure 2: Receiver Operating Characteristic Curves for predicting risk of specific haematological diagnoses in CopLab, WDK and UKB within six months, one year and three years	27](#_Toc234612029)

# Supplementary methods

## Primary care population from Eastern Denmark (CopLab)

*The Copenhagen Primary Care Laboratory Database (CopLab*) includes all results from laboratory tests ordered by any primary care physician from the greater Copenhagen area (Copenhagen Municipality and the former Copenhagen County) from 2000 through 2015 (Figure 1). These test results represent the clinical follow-up of 1.3 million unique individuals from a 1.8 million background population amounting to more than 112 million medical test results including blood tests, urine samples, lung function tests, echocardiograms, and electrocardiograms (ECGs)^1^. Entries in the database have been thoroughly curated and re-labelled ensuring a homogenous and precise dataset. We included individuals from *CopLab* with Danish residence (by January 1^st^ of the included sample) who had at least one CBC registered at any time between January 1^st^, 2006, and December 31^st^, 2015. CBCs from 2001 through 2005 were not included in the main analysis to allow for five years of CBC history predating each CBC included in the model. CBCs from 2000 were excluded due to inconsistent labelling of data in that year. We excluded CBCs from individuals under the age of 18 years and from individuals with previous haematological malignancy defined as an entry in the *Danish Cancer Register* with such a diagnosis predating the included CBC, going back the full extension of the *Danish Cancer Register* i.e., from 1943^2^.

## Complete Blood Cell Counts

CBCs were analysed using Siemens (Bayer/Technicon, Munich, Germany) haematology systems. Each CBC measurement consisted of haemoglobin, platelets, and leukocyte counts (no missing values) as well as up to ten differential cell counts most often included on clinicians’ request. Samples were flagged in case of irregularities such as large unstained cells triggering a manual cell count as well as a differential leukocyte count^1,3^, manual counts replacing the automated ones (Supplementary Table 1). For immature cells, missing values were imputed with zeros as immature cells were only counted when present. In case of missing differential cell count, these were labelled missing yes/no.

# Statistical methods: model building

## Sample splitting and exclusions

We started with all complete blood cell count (CBC) measurements performed in adults between 2006-01-01 and 2015-12-31 (N = 3,714,527 observations, n = 889,581 distinct individuals) in the Copenhagen Primary Care Laboratory (CopLab) Database (see flow chart below). We restricted to observations that occurred before any diagnosis (according to the Danish Cancer Register) of haematological cancers whose International Classification of Disease vs. 10 (ICD-10) codes and morphology codes are listed in Supplementary Table 2 (N = 3,628,898 observations, n = 869,361 distinct individuals). We then restricted it to those that had non-missing values of haemoglobin, platelets, and leukocytes (N = 3,399,591 observations, n = 855,529 distinct individuals). Each CBC measurement consisted of up to 13 variables that represent concentrations of different cell types, as listed in Supplementary Table 1. Haemoglobin, platelets and leukocytes were never missing, but the differential count of leukocytes was only triggered by clinician request, or if an abnormality was detected in the basic counts. Thus, the differential counts could be missing, so we created a missingness indicator for the group of “type 2” cell counts. The immature cells were only counted if they were present, so for observations with type 2 counts present, any missing immature cell counts were imputed with zeros. To help the model differentiate haematological malignancy from acute infections or inflammatory disease affecting the CBC count, we also included C-reactive protein (CRP) measurements along with the CBC counts in the list of predictor variables giving us a total of 14.

For each CBC measurement plus CRP, we constructed additional variables representing the history of prior CBC measurements. This was done by looking back from the CBC date for up to five years and taking the average of each lab value within each of the five years, yielding up to five years-worth of past measurements for each variable. We then proceeded to determine, whether a diagnosis was made by including data from the *Cancer Register*, containing all cancer diagnoses made on individuals with a Danish residence since 1943 ^2^. In this sample, our three outcomes of interest were incident diagnosis of a haematological cancer within six months, one year, and three years (haematological diagnoses from the Cancer Register, Supplemental Table 2). By construction of our sample and since we had complete follow-up in the registers up until 2021-12-31, these outcomes were not subject to right censoring.

These sets of observations were split 60:40 into training and validation subsets based on the Central Persons Register (CPR) unique personal identifier code and these two datasets were from then on kept separate. Sample splitting was done randomly based on personal identifier to reduce bias and overfitting due to the same individual appearing in training and validation^4^. 513,318 (60%) individuals were allocated to the training subsample, while the remaining 342,211 were allocated to the validation subsample. Within the validation subsample, 40,000 individuals were allocated for model recalibration, and the remaining were left for the validation.

From the observations in the training set, we constructed three matched case-control samples, one for each of six months, one year and three years. To do this, for individuals with the outcome present, we identified the CBC observations that were measured within six, 12, and 36 months of the cancer event (N = 4,133 for six months; 5,895 for one year, and 11,779 for three years, CBCs included on average taken 2.1, 4.2 and 14.0 months before the diagnosis), and for each of those observations, randomly selected up to five observations on the same date, without the outcome present, and for an individual of the same sex, and four years or less in age difference. This led to a total of N = 23,150 observations for six months, N = 32,314 for one year, and N = 58,905 for three years comprising 28,348 distinct individuals.

## Figure: Flow chart of sample splitting for model training and internal validation

All eligible CopLab individuals were divided 60:40 into training and validation sub-sets. In the training sub-set, for all individuals with the outcome present (haematological malignancy diagnosed between 2006-01-01 and 2018-31-01) CBCs taken within six months, one year and three years of the diagnosis were matched with up to five CBCs taken on the same date stemming from an individual of the same sex and the same age +/- four years without the outcome present. The three models (six months, one year and three years) were then trained in these three matched sets. Within the validation subset, 40,000 individuals were used for recalibrating the model for the much lower cancer incidence in the validation cohort compared with the matched sets used for training. The remaining individuals in the validation subset was used for model testing. All performance statistics presented in this study were made on the validation subset. CBC = complete blood cell count. Figure created using Biorender.com.

## Model training

We used the *SuperLearner* framework for model training ^5^. Our predictor variables were sex, CRP, age at the CBC measurement, the month of the CBC measurement (to account for seasonal variation), the 13 cell count variables, CRP, and the indicator of non-missing differential cell counts. We also considered models where we additionally included the five years-worth of history of CRP and the CBC measurements. Our outcome variables were the multinomial variables that indicate cancer, death, or emigration within six months, one year, and three years, where zero indicates none of those events. Our library of prediction models included extreme gradient boosted trees with multinomial log loss with the missingness treated as a feature ^6^ and other methods that were preceded by single imputation by predictive mean matching ^7,8^. These latter methods were stepwise variable selection in multinomial regression with a logit link, support vector machines, and the elastic net with alpha set to 0.75, with all pairwise interaction terms and multinomial family with a logit link and the penalty terms selected by internal cross-validation ^9^.

In a ten-fold cross-validation framework, each model in the library was estimated both with and without the history variables and pre-validated predicted probabilities saved for each model. Then two ensemble models, one excluding the history and one including the history, were estimated using the pre-validated ^10^ matrix of eight predictions (four models each without and with the history) by fitting a LASSO penalized binomial regression with the cancer indicators as the outcomes. The final ensemble models are the linear combinations of the individual models in the library fit using all the training data where the coefficients are the optimal ones determined in the ensemble step. When predictor variables are provided to the model, it returns the predicted probabilities of a haematological cancer within six months, one year, and three years.

## Model evaluation

In the hold-out validation dataset, which includes individuals who were not used at all in the model training process, we evaluated and compared the optimal ensemble models with and without the history by estimating receiver operating characteristic (ROC) curves and areas under the ROC curves estimated using the *plotROC R* package ^11^. We also compared our models to the true and false positive fractions of using the World Health Organization criteria as detailed in Supplementary Table 3 and estimated ROC curves and their AUCs separately by cancer subtype. In addition to the ROC curves, we also plotted calibration curves to assess how well the predicted probabilities align with the observed ^12^.

## Recalibration

Since the training data was based on a case-control sample in which the prevalence of the cancer outcomes was much higher than they are in the population of intended use, we needed to recalibrate the model, so the average of the predicted probabilities more closely reflects the marginal probability of the outcome in the population. Thus, using the subsample of 40,000 individuals from the validation set (not a case-control sample), we used logistic recalibration in 5000 randomly selected subsamples containing one observation per individual and then took the average of the estimated slopes and intercepts ^13^.

## Surrogate model estimation

Since the model is extremely complex and potentially contains personal data, we estimated a simpler surrogate model that can be exported from the Statistics Denmark servers and shared publicly. To do this we trained a model using the *Cubist R* package ^14^ with the recalibrated risk scores as the outcome and the same predictors. We did this separately by whether immature cell counts were available or not, and with five rules each. R code to get predictions from this model is available in a later section.

## Feature importance

Feature importance of the model was estimated using the *FeatureImp* function of the *iml R* package ^15^. This fits a xgboost-based surrogate model to the outcome using the same predictors, and then randomly perturbs predictors one-by-one to see the effect on the prediction performance measured by median absolute error. We did this overall and for each cancer type separately and plotted the results for the top five most important features.

## External validations

For the external validation in the Western Denmark region (validation cohort 1), we used the full six-month model exactly as it was trained on the training sample to obtain predicted risk values for each laboratory measurement in the sample. We also used the surrogate model in the same way for comparison and to ensure that predictive power was not lost. For the external validation in the UKBB, we could only apply the surrogate model as it was outside of Statistics Denmark’s secure servers.

## Cumulative incidence according to predicted risk

We modelled the cumulative incidence of haematological malignancy according to predicted risk with death and emigration as competing risks in both an unadjusted analysis and adjusted for demographics and co-morbidities using pseudo-observation-based regression models^16^. This was done to evaluate how risk predictions aligned with observed disease incidence over time. To adjust for comorbidities in this analysis, we included information on co-morbidities (Supplementary Table 4) indexed in the *National Patient Register^17^* occurring within 5 years of each CBC, labelling each disease as present yes/no.

# Statistical methods: comparison with standard of care

## Model – true positives, false positives and predictive values

We estimated performance, proportion test positive, true and false positive fractions, and positive predictive values (PPV) at different risk prediction thresholds of our model by running multiple sequential cohort analyses beginning with three-month time intervals; cohort 1 commencing on January 1^st^, 2001, a new cohort starting every three months until our study period ended on December 31^st^, 2015. Eligibility was assessed at the beginning of each cohort, CopLab participants over the age if 18 years with no prior haematological diagnosis defined as a diagnosis made more than three months before time zero were included and followed for three months. In each cohort, we looked at the first CBC measurement available for each participant during that three-month interval, calculated the risk score, and then looked forward six months from the CBC measurement for any haematological cancer diagnosis. If an individual had no CBC during that time interval, they were not included in that cohort but could be eligible again for the next cohort. We did this for each cohort study, estimating the true and false positive fractions and PPVs, and NPVs for all possible risk score thresholds, bootstrap resampling individuals to calculate 95% confidence intervals.

## Standard of care – true positives, false positives and predictive values

To compare the model performance with standard of care, we needed to ascertain the proportion test positive, true and false positive fractions and PPV for the current approach: clinical evaluation by a primary care physician in Denmark. To do this, we needed information for a standard two-by-two table:

|  | No, cancer | Yes, cancer | TOTAL |
| --- | --- | --- | --- |
| Negative test | True negatives | False negatives |  |
| Positive test | False positives | True positives |  |
| TOTAL |  |  |  |

The ‘test’ under evaluation in our case is the clinical decision-making process of the primary care physician. So, a positive test was defined as a referral to haematological care. The presence of a haematological cancer diagnosis settled the division into cancer yes/no. The false positives would be referrals that did not lead to a cancer diagnosis and true positives would be a referral that resulted in a cancer diagnosis. A negative test i.e., the decision *not* to refer a patient was defined as no referral present in that three-month time interval (see below).

We proceeded to gather data on actual visits in haematological departments in Danish hospitals. Data registered in the secondary sector is indexed in the *National Patient Register (NPR)*, a unique nationwide register containing detailed health data including ICD-10 codes, hospital admissions and visits to outpatient clinics on every individual with permanent residence in Denmark going back to 1977 ^18^. Reporting to *the NPR* is a part of the billing system for hospitals in Denmark and diagnostic as well as procedure codes and hospital unit codes are therefore considered complete and ICD-10 codes are continuously validated ^19^. In *the NPR* information on hospital admittance and visits at outpatient clinics are thoroughly registered on a national scale for all public and private hospitals. The data includes dates of visits and hospital admittance as well as diagnoses, procedure codes and data detailing the departments involved and their medical specialty. We extracted all first-time visits registered for our CopLab individuals in any Danish department of haematology - both current and historic departments active during the CopLab inclusion. However, *the NPR* only registers actual visits in the secondary sector, whereas the referrals leading to the visits are not registered. We were quite certain that a considerable proportion of referrals to haematological clinics are rejected by specialists without a visit and these patients are thus not registered in *the NPR* – typically due to referral criteria not being met or relevant work-up lacking from primary care*.* The rejected referral are, however, relevant when quantifying the current standard of care: we are interested in referrals rather than visits for comparison with the model predictions, as the model is validated for use in primary care for guiding the decision to refer - and not in the haematological clinics to guide the decision to accept or decline the referral. So, we contacted the haematological department at the Copenhagen University Hospital, Rigshospitalet, which is the largest haematological department in Denmark and responsible for about half of the haematological patients with samples in CopLab. They provided us with clinical quality assurance data from 2018-2022, detailing the decisions made for each referral received from primary care. The department receives referrals into two separate teams: a lymphoid and a myeloid team and on average these two teams reject 39 % of the referrals they receive from primary care. We find it safe to assume that this rejection fraction is representative for the clinical practice in all the region as both regional hospitals are highly specialized and follow the same guidelines for rejecting and accepting referrals. Then for every haematological visit registered in *the NPR*, there are 1/0.39 referrals that are declined, and the total number of referrals will equal the number of visits registered in *the NPR**(1/0.39).

We then proceeded to carry out sequential cohort analyses starting with three-month time intervals, cohort 1 commencing on 01-01-2001. Eligibility was assessed at the beginning of each interval following the same criteria as described in the above. Each eligible individual was followed for three months looking for a first time visit in haematology. If no visit occurred during the three-month interval, it was registered as a negative test. The resulting number of positive tests i.e., visits were then multiplied by 1/0.39 to account for rejected referrals not resulting in a visit to a haematological department (still a positive test but not registered in *the NPR*). We included haematological diagnoses registered up to one month before or six months after the visit to account for any delay in the diagnostic process following a visit in haematology. Diagnoses occurring up to 1 month before the visit were included because lymphoma patients are most often diagnosed by biopsy before entering the Danish haematological clinics. When defining eligibility, we chose to include patients who had a haematological diagnosis within three months of time zero to account for these procedures for lymphoma patients. If no visit was registered i.e., a negative test, we looked ahead six months from the beginning of the three-month time interval for a haematological diagnosis, if such diagnosis occurred we labelled the test a false negative, however, this is does not accurately reflect clinical practice as you cannot get a haematological diagnosis without testing at a haematological clinic, however if the diagnosis occurred more than six months after a visit in haematology, it would be logged in the false negative category.

To get 95% confidence intervals on the measures of performance that account for the multiple observations per individual, we used the nonparametric bootstrap by resampling individuals (rather than observations).

# Results

## Performance of the full ensemble model

*CBC-HEMA* was created based on a supervised stacked machine learning model, which was however not exportable due to legislation protecting personal information, which could be deduced from the model. The full ensemble model predicting haematological malignancy in CopLab validation cohort participants within six months, one year and three years yielded AUCs of 0.86, 0.81 and 0.76, respectively. Adding the five-year CBC history did not markedly improve the model’s performance and the surrogate model, CBC-HEMA based on one CBC measure also performed comparably well with AUCs of 0.81, 0.78 and 0.71 for predicting overall haematological malignancy within six months, one year and three years, respectively.

The full six-month ensemble model validated well in WDK with AUCs of 0.81, 0.78 and 0.71 within six months, one-year and three-years, respectively. *CBC-HEMA* performed close to the full ensemble model with AUCs of 0.77, 0.75, and 0.68 within six months, one-year, and three-years, respectively. In UKB *CBC-HEMA* performed with AUCs of 0.67, 0.65 and 0.59 for predicting overall haematological malignancy within six months, one year and three years, respectively.

Looking at the calibration curves (Supplementary Figure 1) presenting the average observed risk for bins of predicted risks, the ensemble model appeared exceedingly well calibrated in CopLab with a slope of 0.993 and an intercept of -0.42, while the surrogate, *CBC-HEMA*, appeared to mildly underestimate the risk of haematological malignancy across most threshold probabilities (slope 1.18 and intercept 0.43). Similar metrics were achieved in WDK for the ensemble model, while *CBC-HEMA* with a slope of 1.36 had a slightly greater tendency for underestimating risk than in CopLab. Data sparsity resulted in an unstable curve for the UKB calibration, and the slope and intercepts should be interpreted with caution, but we generally consider calibration in UKB to be inferior to WDK. This translates to a risk that the model – if implemented in the clinic for use independent of clinician oversight - would overlook a small proportion of patients and *CBC-HEMA* should only be used in concert with clinical assessment.

## Risk stratification using CBC-HEMA

We analysed the observed cumulative incidence of haematological malignancy according *to* *CBC-HEMA* predicted risks with death and emigration as competing risks defining three separate risk groups: A low-risk group defined by a threshold of 2.25 % and dividing the remaining observations in an intermediate and high-risk group separated by the median (5 %) (see figure below). Disease incidence was markedly different for these three risk groups, who appear to represent clinically separate groups. No change when adjusting for demographics and co-morbidity (table below, adjusted distributions not shown). This observation suggests that the relationship between predicted risk and observed incidence of malignancy is independent of co-morbidity and demographics.

# Figure: Cumulative incidence of any type of haematological malignancy by predicted risk score in the CopLab, WDK-and UKB-populations

CopLab = Copenhagen Primary Care Laboratory Database. WDK= Western Denmark. UKB = UK Biobank. CBC = complete blood cell count. Cumulative incidence of any haematological malignancy following a CBC with death and emigration as competing risks in the internal validation cohort from CopLab and in external validation cohorts: WDK consisting of primary care patients from Western Denmark and UKB consisting of volunteers from the British background population. For CopLab patients, cumulative incidence was adjusted for demographics and for demographics and co-morbidity in the form of 12 binary variables of disease labelled as present yes/no within five years of each CBC. These included cancer, cerebrovascular disease, chronic pulmonary disease, congestive heart failure, dementia, rheumatic diseases, diabetes, liver disease, myocardial infarction, peripheral vascular disease, and renal disease (table below) indexed in the Danish *National Patient Register.*

# Table: ICD-10 codes for co-morbidity

| Disease group | ICD-10 diagnosis |
| --- | --- |
| Cardiovascular disease | DI21; DI22; DI252 |
| Congestive heart failure | DI43; DI50; DI099; DI110; DI130; DI132; DI255; DI420; DI425; DI426; DI427; DI428; DI429; DP290 |
| Peripheral vascular disease | DI70; DI71; DI731; DI738; DI739; DI771; DI790; DI792; DK551; DK558; DK559; DZ958; DZ959 |
| Cerebrovascular disease | DG45; DG46; DI60; DI61; DI62; DI63; DI64; DI65; DI66; DI67; DI68; DI69; DH340 |
| Dementia | DF00; DF01; DF02; DF03; DG30; DF051; DG311 |
| Chronic pulmonary disease | DJ40; DJ41; DJ42; DJ43; DJ44; DJ45; DJ46; DJ47; DJ60; DJ61; DJ62; DJ63; DJ64; DJ65; DJ66; DJ67; DI278; DI279; DJ684; DJ701; DJ703 |
| Connective tissue disease and rheumatic disease | DM05; DM32; DM33; DM34; DM06; DM315; DM351; DM353; DM360 |
| Peptic ulcer disease | DK25; DK26; DK27; DK28 |
| Mild liver disease | DB18; DK73; DK74; DK700; DK701; DK702; DK703; DK709; DK717; DK713; DK714; DK715; DK760; DK762; DK763; DK764; DK768; DK769; DZ944 |
| Moderate or severe liver disease | DK704; DK711; DK721; DK729; DK765; DK766; DK767; DI850; DI859; DI864; DI982 |
| Diabetes without complications | DE100; DE101; DE106; DE108; DE109; DE110; DE111; DE116; DE118; DE119; DE120; DE121; DE126; DE128; DE129; DE130; DE131; DE136; DE138; DE139; DE140; DE141; DE146; DE148; DE149 |
| Diabetes with complications | DE102; DE103; DE104; DE105; DE107; DE112; DE113; DE114; DE115; DE117; DE122; DE123; DE124; DE125; DE127; DE132; DE133; DE134; DE135; DE137; DE142; DE143; DE144; DE145; DE147 |
| Paraplegia and hemiplegia | DG81; DG82; DG041; DG114; DG801; DG802; DG830; DG831; DG832; DG833; DG834; DG839 |
| Renal disease | DN18; DN19; DN052; DN053; DN054; DN055; DN056; DN057; DN250; DI120; DI131; DN032; DN033; DN034; DN035; DN036; DN037; DZ490; DZ491; DZ492; DZ940; DZ992 |
| Cancer | DC00; DC01; DC02; DC03; DC04; DC05; DC06; DC07; DC08; DC09; DC10; DC11; DC12; DC13; DC14; DC15; DC16; DC17; DC18; DC19; DC20; DC21; DC22; DC23; DC24; DC25; DC26; DC30; DC31; DC32; DC33; DC34; DC37; DC38; DC39; DC40; DC41; DC43; DC45; DC46; DC47; DC48; DC49; DC50; DC51; DC52; DC53; DC54; DC55; DC56; DC57; DC58; DC60; DC61; DC62; DC63; DC64; DC65; DC66; DC67; DC68; DC69; DC70; DC71; DC72; DC73; DC74; DC75; DC76; DC81; DC82; DC83; DC84; DC85; DC88; DC90; DC91; DC92; DC93; DC94; DC95; DC96; DC97 |
| Metastatic carcinoma | DC77; DC78; DC79; DC80 |
| AIDS/HIV | DB20; DB21; DB22; DB24 |

ICD-10 = International Classification of Disease, version 10.

## Sensitivity, Specificity, Positive and Negative Predictive Values

Using the methods outlined in the section on *Statistical methods – comparison with standard of care*, we arrived at the following results:

|  | Sensitivity | Specificity | PPV | NPV |
| --- | --- | --- | --- | --- |
| Standard of Care | 0.94 (0.93;0.95)* | 0.99 (0.99;0.99) | 0.21 (0.21;0.22) | 1.00 (1.00;1.00) |
| WHO 2016 | 0.54 (0.54;0.56) | 0.72 (0.72;0.72) | 0.0027 (0.003;0.003) | 0.999 (0.999;0.999) |
| WHO 2022 | 0.69 (0.68;0.70) | 0.65 (0.35;0.36) | 0.0027 (0.002;0.003) | 0.999 (0.999;0.999) |

* (95 % confidence interval), all such numbers. PPV = positive predictive value. NPV = negative predictive value. WHO 2016 and 2022 = simple laboratory cutoffs represented by laboratory values referenced in the world health organisation guidelines for diagnosing haematological malignancies from 2016 and 2022.

# Figure: Number of diagnoses and complete blood cell counts in CopLab by calendar year

Number of CBCs and individuals in CopLab according to calendar year (left). Number of malignant haematological diagnoses and number of first-time visits at haematological clinics per calendar year (right).

# Figure: Cumulative incidence of haematological malignancy with fixed axes.

CopLab = Copenhagen Primary Care Laboratory Database. WDK= Western Denmark. UKB = UK Biobank. CBC = complete blood cell count. A: Histogram of age distribution in the combined CopLab cohort separate subsets of which was used to train and internally validate the model and in external validation cohorts: WDK consisting of primary care patients from Western Denmark and UKB consisting of volunteers from the British background population. B: Cumulated incidence of haematologicalal malignancy and death in CopLab, WDK and UKB.

# References

1.	Kriegbaum M, Lind BS, Grand MK, Andersen CL. The Copenhagen Primary Care Laboratory (CopLab) Database. *Clin Epidemiol*. 2024;16:155-163. doi:10.2147/CLEP.S437123

2.	Gjerstorff ML. The Danish Cancer Registry. *Scand J Public Health*. 2011;39(7):42-45. doi:10.1177/1403494810393562

3.	Andersen CL, Siersma VD, Karlslund W, et al. The Copenhagen primary care differential count (CopDiff) database. *Clin Epidemiol*. 2014;6(1):199-211. doi:10.2147/CLEP.S60991

4.	Coley RY, Walker RL, Cruz M, Simon GE, Shortreed SM. Clinical risk prediction models and informative cluster size: Assessing the performance of a suicide risk prediction algorithm. *Biometrical Journal*. 2021;63(7):1375-1388. doi:10.1002/bimj.202000199

5.	Van Der Laan MJ, Polley EC, Hubbard AE. Super learner. *Stat Appl Genet Mol Biol*. 2007;6(1). doi:10.2202/1544-6115.1309

6.	Chen T, Guestrin C. XGBoost: A scalable tree boosting system. *Proceedings of the ACM SIGKDD International Conference on Knowledge Discovery and Data Mining*. 2016;13-17-Augu:785-794. doi:10.1145/2939672.2939785

7.	van Buuren S, Groothuis-Oudshoorn K. mice: Multivariate imputation by chained equations in R. *J Stat Softw*. 2011;45(3):1-67. doi:10.18637/jss.v045.i03

8.	Little RJA, Rubin DB. *Statistical Analysis with Missing Data*. John Wiley & Sons, Ltd; 2019.

9.	Friedman J, Hastie T, Tibshirani R. Regularization paths for generalized linear models via coordinate descent. *J Stat Softw*. 2010;33(1):1-22. doi:10.18637/jss.v033.i01

10.	Tibshirani RJ, Efron B. Pre-validation and inference in microarrays. *Stat Appl Genet Mol Biol*. 2002;1(1). doi:10.2202/1544-6115.1000

11.	Sachs MC. Plotroc: A tool for plotting ROC curves. *J Stat Softw*. 2017;79(August):1-19. doi:10.18637/jss.v079.c02

12.	Harrell FE. *Regression Modelling Strategies*. Springer New York; 2001.

13.	Steyerberg EW. *Clinical Prediction Models: A Practical Approach to Development, Validation and Updating*. Springer; 2019.

14.	Kuhn M, Quinlan R, Weston S, Keefer C, Coulter N. Cubist: Rule- And Instance-Based Regression Modeling. R package version 0.4.2.1. 2023. https://cran.r-project.org/package=Cubist

15.	Molnar C. iml: An R package for Interpretable Machine Learning. *J Open Source Softw*. 2018;3(26):786. doi:10.21105/joss.00786

16.	Sachs MC, Gabriel EE. Event History Regression with Pseudo-Observations: Computational Approaches and an Implementation in R. *J Stat Softw*. 2022;102(9). doi:10.18637/jss.v102.i09

17.	Lynge E, Sandegaard JL, Rebolj M. The Danish national patient register. *Scand J Public Health*. 2011;39(7):30-33. doi:10.1177/1403494811401482

18.	Lynge E, Sandegaard JL, Rebolj M. The Danish national patient register. *Scand J Public Health*. 2011;39(7):30-33. doi:10.1177/1403494811401482

19.	Schmidt M, Schmidt SAJ, Sandegaard JL, Ehrenstein V, Pedersen L, Sørensen HT. The Danish National patient registry: A review of content, data quality, and research potential. *Clin Epidemiol*. *Dove Press*. 2015;7:449-490. doi:10.2147/CLEP.S91125

20.	Alaggio R, Amador C, Anagnostopoulos I, et al. The 5th edition of the World Health Organization Classification of Haematolymphoid Tumours: Lymphoid Neoplasms. *Leukemia 2022 36:7*. 2022;36(7):1720-1748. doi:10.1038/s41375-022-01620-2

21.	Khoury JD, Solary E, Abla O, et al. The 5th edition of the World Health Organization Classification of Haematolymphoid Tumours: Myeloid and Histiocytic/Dendritic Neoplasms. *Leukemia*. 2022;36(7):1703-1719. doi:10.1038/s41375-022-01613-1

22.	Arber DA, Orazi A, Hasserjian R, et al. The 2016 revision to the World Health Organization classification of myeloid neoplasms and acute leukemia. *Blood*. 2016;127(20):2391-2405. doi:10.1182/blood-2016-03-643544

23.	Swerdlow SH, Campo E, Pileri SA, et al. The 2016 revision of the World Health Organization classification of lymphoid neoplasms. *Blood*. 2016;127(20):2375-2390. doi:10.1182/blood-2016-01-643569

# R code for the model

calc_risk_cbc_hema <- function(AgeAtTest, testmonth, genderM, CRP,

HB, LEUK, TRC,

BASO, BLAST, EOS, LYMF, META,

MONO, MYEL, NEUT,

PROM, UKLAS){

META[is.na(META)] <- 0

MYEL[is.na(MYEL)] <- 0

PROM[is.na(PROM)] <- 0

UKLAS[is.na(UKLAS)] <- 0

\## type 1 blood test -- only HB, LEUK and TRC available

type1_logi <- (is.na(BASO) | is.na(BLAST) | is.na(EOS) | is.na(LYMF) |

is.na(META) | is.na(MONO) | is.na(MYEL) | is.na(NEUT) |

is.na(PROM) | is.na(UKLAS))

\## impute missing CRPS

CRP[is.na(CRP) & type1_logi] <- 5

CRP[is.na(CRP) & !type1_logi] <- 5

lrs_type1 <-

ifelse(LEUK <= 10.1 & TRC <= 467 & CRP <= 7.7,

(-6.2924817) - (0.0201 * CRP) - (0.126 * HB) + (0.028 * LEUK) + (0.0004 * TRC) + (0.04 * genderM) + (0.001 * AgeAtTest),

ifelse(LEUK <= 10.1 & CRP > 7.7,

(-5.2361444) - (0.228 * HB) + (0.00078 * TRC) + (0.1 * genderM) - (0.0023 * AgeAtTest) + (0.0018 * CRP) + (0.008 * LEUK),

ifelse(LEUK > 10.1,

(-6.643347) + (0.159 * LEUK) - (0.233 * HB) + (0.08 * genderM) + (0.0005 * AgeAtTest),

ifelse(HB <= 7,

(-1.1491499) - (0.824 * HB) + (0.48 * genderM) - (0.017 * testmonth) + (0.00068 * TRC) - (0.0027 * AgeAtTest) + (0.011 * LEUK) + (0.0009 * CRP),

ifelse(TRC > 467,

(-15.9967071) + (0.01249 * TRC) + (0.357 * HB) + (0.047 * testmonth),

NA

)

)

)

)

)

lrs_type2 <- ifelse(TRC <= 475,

(-5.8901762) + (16.69 * MYEL) + (0.068 * LEUK) - (0.187 * HB) - (0.067 * LYMF) + (0.001 * TRC) + (0.004 * CRP) + (0.12 * genderM) - (0.025 * NEUT) + (0.16 * MONO) - (0.15 * EOS) - (0.0011 * AgeAtTest) + (0.3 * BASO),

ifelse(LEUK > 9.6 & TRC <= 383,

(-5.5542763) + (0.145 * LEUK) - (0.313 * HB) - (0.107 * NEUT) + (0.0024 * TRC) + (1.2 * MYEL) + (0.21 * genderM) - (0.0045 * AgeAtTest) + (0.038 * LYMF) + (0.0015 * CRP) + (0.7 * BASO) + (0.1 * MONO),

ifelse(HB <= 7.5,

(-1.6094342) - (0.398 * LEUK) + (0.38 * NEUT) - (0.704 * HB) + (0.409 * LYMF) + (0.51 * genderM) + (0.93 * MONO) + (0.42 * BLAST) + (0.0059 * CRP) - (0.0061 * AgeAtTest) + (0.0011 * TRC) + (0.47 * MYEL) + (1.7 * BASO) - (0.29 * EOS) + (0.6 * UKLAS) - (0.007 * testmonth) + (0.5 * META),

ifelse(LEUK > 9.6 & TRC > 383,

(-10.4425541) + (0.484 * LYMF) + (0.0085 * TRC) + (0.309 * NEUT) - (0.151 * LEUK) - (0.015 * AgeAtTest) - (0.17 * HB) + (0.3 * genderM) + (1.7 * UKLAS) + (0.57 * MONO) + (0.49 * EOS) + (1.9 * BASO) - (0.007 * testmonth),

ifelse(TRC > 475,

(-14.2218279) + (0.0111 * TRC) + (0.271 * LEUK) - (0.507 * LYMF) - (0.235 * NEUT) + (0.497 * HB) - (1.64 * MONO) - (0.0163 * AgeAtTest) + (1.9 * UKLAS) - (0.69 * EOS) + (0.027 * testmonth) + (1.8 * BASO) + (0.08 * genderM) + (0.0019 * CRP),

NA

)

)

)

)

)

plogis(ifelse(type1_logi, lrs_type1, lrs_type2))

}

# Supplementary Table 1: Catalogue of biochemical analyses included in the complete blood cell count in the CopLab database

| Basic analysis | NPU | Min | Max |
| --- | --- | --- | --- |
| B-Haemoglobin | 02319 | 0 | 14.0 |
| B-Platelets | 03568 | 0 | 2200 |
| B-Leukocytes | 02593 | 0 | 650 |
| Differential cell counts |  |  |  |
| B-Lymphocytes | 02636 |  |  |
| B-Neutrophils | 02902 |  |  |
| B-Monocytes | 02840 |  |  |
| B-Eosinophils | 01933 |  |  |
| B-Basophils | 01349 |  |  |
| Immature Cells^1^ |  |  |  |
| B-Blasts | 03972 |  |  |
| B-Myelocytes | 03976 |  |  |
| B-Metamyelocytes | 03978 |  |  |
| B-Promyelocytes | 03974 |  |  |
| B-Unclassifiable^2^ |  |  |  |

CopLab = Copenhagen Primary Care Laboratory Database. NPU = nomenclature for properties and units code. Min: minimum value recorded in CopLab. Max = maximum value recorded in CopLab. ^1^Immature leukocytes are classified in manual counts. ^2^Cells unclassifiable in the automated count, the presence of which will trigger a manual re-count, replacing the automated count.

# Supplementary Table 2: ICD-10 diagnoses included within each diagnostic subgroup

|  | Haematological malignancy |  |
| --- | --- | --- |
| Disease group | **ICD-10 diagnosis** | **ICD-O-3 codes^1^** |
| Hodgkin lymphoma | C81 Hodgkin lymphoma | 9650-9653, 9659, 9663 |
| Non-Hodgkin lymphoma (NHL) | C82 Follicular lymphoma  C83 Non-follicular lymphoma  C84 Mature T/NK-cell lymphomas  C85 Other and unspecified types of non-Hodgkin lymphoma  C88.3 Immunoproliferative small intestinal disease  C88.4 Extranodal marginal zone B-cell lymphoma of mucosa associated lymphoid tissue (MALT-lymphoma)  C88.7 Other malignant immunoproliferative diseases  C88.9 Malignant immunoproliferative disease, unspecified | 9591, 9596-9597, 9671, 9673, 9678-9680, 9687-9690, 9699, 9702, 9705, 9714, 9716-9717, 9735, 9737-9738, 9766 |
| Plasma Cell Disease (PCD) | C88.0 Waldenströms macroglobulinemia  C88.2 Other heavy chain disease  C90 Multiple myeloma and malignant plasma cell neoplasms  D47.2 Monoclonal gammopathy of undetermined potential (MGUS) |  |
| Chronic lymphoid leukaemia (CLL) | C91.1 Chronic lymphoid leukaemia of B-cell type  C91.3 Prolymphocytic leukaemia of B-cell type  C91.4 Hairy cell leukaemia  C91.6 Prolymphocytic leukaemia of T-cell type  C91.7 Other lymphoid leukaemia  C91.9 Lymphoid leukaemia, unspecified |  |
| Acute lymphoblastic leukaemia (ALL) | C91.0 Acute lymphoblastic leukaemia  C91.8 Mature B-cell leukaemia, Burkitt type |  |
| Myeloproliferative neoplasms (MPN) | C92.1 Chronic myeloid leukaemia, BCR/ABL-positive  C92.2 Atypical chronic myeloid leukaemia, BCR/ABL-negative  D45 Polycythaemia Vera  D47.0 Histiocytic and mast cell tumours of uncertain or unknown behaviour  D47.1 Chronic myeloproliferative disease  D47.3 Essential thrombocythemia  D47.4 Osteomyelofibrosis  D47.5 Chronic eosinophilic leukaemia |  |
| Myelodysplastic neoplasms (MDS) | D46 Myelodysplastic syndromes  C93.1 Chronic myelomonocytic leukaemia  C93.2 Juvenile myelomonocytic leukaemia  C93.9 Monocytic leukaemia, unspecified |  |
| Acute myeloid leukaemia (AML) | C92.0 Acute myeloblastic leukaemia  C92.3 Myeloid sarcoma  C92.4 Acute promyelocytic leukaemia  C92.5 Acute myelomonocytic leukaemia  C92.6 Acute leukaemia with 11q23-abnormality  C92.8 Acute myeloid leukaemia with multilineage dysplasia  C93.0 Acute monoblastic/monocytic leukaemia  C94.0 Acute erythroid leukaemia  C94.2 Acute megakaryoblastic leukaemia  C94.3 Mast cell leukaemia  C94.4 Acute panmyelosis with myelofibrosis |  |

ICD-10 = International Classification of Disease, version 10.

^1^ICD-O-3 codes for lymphoma were initially included but then replaced by the ICD-10 diagnosis in the dataset if such a diagnosis was registered. In cases with no ICD-10 diagnosis, the ICD-O-3 code was used for classification into Hodgkin and Non-Hodgkin lymphoma as described.

# Supplementary Table 3: WHO 2022 and WHO 2016 laboratory criteria included for comparison

| WHO 2022 | WHO 2016 |
| --- | --- |
| Haemoglobin ≥ 9.9 mmol/L for women and ≥ 10.2 mmol/L for men | Haemoglobin ≥ 9.9 mmol/L for women and ≥ 10.2 mmol/L for men |
| Platelet count ≥ 450 x 10^9^/L for 3 months | Platelet count ≥ 450 x 10^9^/L for 3 months |
| Leukocytes ≥ 13 x 10^9^/L and platelet count ≥ 450 x 10^9^/L | Leukocytes ≥ 11 x 10^9^/L and platelet count ≥ 450 x 10^9^/L |
| Monocytes ≥ 0.5 x 10^9^/L comprising ≥ 10% of total WBC for 3 months | Monocytes ≥ 1.0 x 10^9^/L comprising ≥ 10% of total WBC for 3 months |
| Lymphocytes ≥ 5 x 10^9^/L for more than 3 months | Lymphocytes ≥ 5 x 10^9^/L for more than 3 months |
| Leukocytes ≥ 25 x 10^9^/L | Leukocytes ≥ 25 x 10^9^/L |
| Cytopenia:   - Haemoglobin < 8,0 (M) and 7,4 (F) mmol/L, or - Platelet count < 150 x 10^9^/L, or - Absolute neutrophil count < 1.8 x 10^9^/L. | Cytopenia   - Haemoglobin < 6.2 mmol/L, or - Platelet count < 100 x 10^9^/L, or - Absolute neutrophil count < 1.8 x 10^9^/L. |
| Blasts in peripheral blood > 0.0 x 10^9^/L | Blasts in peripheral blood > 0.0 x 10^9^/L |

WHO = world health organization ^20–23^. WBC = white blood cell count.

# Supplementary Figure 1: Calibration curves for predicting any haematological malignancy

Full 6m model = ensemble model for 6-months predictions*.* Calibration curve showing the average observed risks according to bins of predicted risks. The referral threshold tested in CopLab was a predicted risk of 0.0225. Slope and intercept for CopLab: full 6m ensemble model: 0.993 and -0.42. Slope and intercept, *CBC-HEMA*: 1.18 and 0.42. WDK, ensemble, slope and intercept: 0.90 and -0.45. WDK, *CBC-HEMA*, slope and intercept: 1.36 and 0.86. UKB, *CBC-HEMA*: slope = 1,24, intercept = 0.46.

# Supplementary Figure 2: Receiver Operating Characteristic Curves for predicting risk of specific haematological diagnoses in CopLab, WDK and UKB within six months, one year and three years

CopLab = Copenhagen Primary Care Laboratory Database. WDK= Western Denmark. UKB = UK Biobank. ROC = receiver operations characteristics curve. AUC = area under the curve. AML = acute myeloid leukaemia. MDS = myelodysplastic neoplasms. MPN = myeloproliferative neoplasms. ALL = acute lymphoblastic. Receiver Operating Characteristic (ROC) curves for predicting myeloid haematological malignancies and Acute Lymphoid Leukaemia within six months (red), one year (green) and three years (blue) following a CBC in the internal validation cohort from CopLab and in external validation cohorts: WDK consisting of primary care patients from Western Denmark and UKB consisting of volunteers from the British background population. The CopLab cohort used to train the model was kept separate from the internal validation cohort, model performance in the training subset is not reported.
